# Stimuli-Responsive Codelivery System-Embedded Polymeric Nanofibers with Synergistic Effects of Growth Factors and Low-Intensity Pulsed Ultrasound to Enhance Osteogenesis Properties

**DOI:** 10.1021/acsabm.4c00111

**Published:** 2024-06-25

**Authors:** Samira Malekmohammadi, Rashid Jamshidi, Joanna M. Sadowska, Chen Meng, Chamil Abeykoon, Mohsen Akbari, R. Hugh Gong

**Affiliations:** † Department of Materials, Engineering Building A, University of Manchester, Manchester M13 9PL, U.K.; ‡ Department of Engineering, Manchester Metropolitan University, Manchester M1 5GD, U.K.; § Advanced Materials and Bioengineering Research Centre (AMBER), Royal College of Surgeons in Ireland and Trinity College Dublin, Dublin D02 YN77, Ireland; ∥ Tissue Engineering Research Group, Department of Anatomy & Regenerative Medicine, Royal College of Surgeons in Ireland, Dublin D02 YN77, Ireland; ⊥ Laboratory for Innovations in Microengineering (LiME), Department of Mechanical Engineering, University of Victoria, Victoria, British Columbia V8P 5C2, Canada; # Terasaki Institute for Biomedical Innovations, Los Angeles, California 90024, United States

**Keywords:** drug delivery, low-intensity pulsed ultrasound, controlled release, bone tissue regeneration, dendritic silica nanoparticles, scaffold

## Abstract

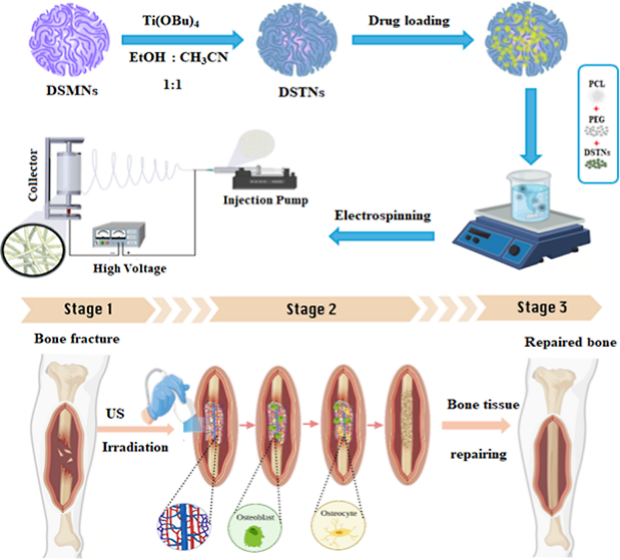

The present work aims to develop optimized scaffolds for bone repair by incorporating mesoporous nanoparticles into them, thereby combining bioactive factors for cell growth and preventing rapid release or loss of effectiveness. We synthesized biocompatible and biodegradable scaffolds designed for the controlled codelivery of curcumin (CUR) and recombinant human bone morphogenic protein-2 (rhBMP-2). Active agents in dendritic silica/titania mesoporous nanoparticles (DSTNs) were incorporated at different weight percentages (0, 2, 5, 7, 9, and 10 wt %) into a matrix of polycaprolactone (PCL) and polyethylene glycol (PEG) nanofibers, forming the CUR-BMP-2@DSTNs/PCL–PEG delivery system (S0, S2, S5, S7, S9, and S10, respectively, with the number showing the weight percentage). To enhance the formation process, the system was treated using low-intensity pulsed ultrasound (LIPUS). Different advanced methods were employed to assess the physical, chemical, and mechanical characteristics of the fabricated scaffolds, all confirming that incorporating the nanoparticles improves their mechanical and structural properties. Their hydrophilicity increased by approximately 25%, leading to ca. 53% enhancement in their water absorption capacity. Furthermore, we observed a sustained release of approximately 97% for CUR and 70% for BMP-2 for the S7 (scaffold with 7 wt % DSTNs) over 28 days, which was further enhanced using ultrasound. In vitro studies demonstrated accelerated scaffold biodegradation, with the highest level observed in S7 scaffolds, approximately three times higher than the control group. Moreover, the cell viability and proliferation on DSTNs-containing scaffolds increased when compared to the control group. Overall, our study presents a promising nanocomposite scaffold design with notable improvements in structural, mechanical, and biological properties compared to the control group, along with controlled and sustained drug release capabilities. This makes the scaffold a compelling candidate for advanced bone tissue engineering and regenerative therapies.

## Introduction

Bone tissues possess a unique self-repair capacity in response to minor trauma or injuries. However, severe fractures, diseases, or tumors frequently generate critical-sized or nonunion defects, which reduce the self-healing capacity of the bone; therefore, bone grafts are required to support and stimulate the healing process. Such grafts are usually synthetic biomaterial scaffolds, which may be based on polymers,^1−5^ ceramics,^6−10^ metals,^11,12^ or composites,^13−16^ as all of these have low risks of immunogenicity, and unlike autografts and allografts they are widely accessible. For instance, polycaprolactone (PCL) and polyethene glycol (PEG) are Food and Drug Administration (FDA) approved synthetic polymers with a proven history in biomedical applications, having favorable biocompatible and biodegradable properties, as well as being easily processed into desired shapes using additive manufacturing or electrospinning techniques.^17,18^ PCL can be blended with PEG, leading to more favorable rheological and adhesive properties than PCL alone.^19,20^ Furthermore, the incorporation of PEG into PCL provides greater thermoresistance of the scaffolds as well as enables prolonged, sustained, and controlled release of therapeutic molecules.^21,22^ Altogether, this combination has resulted in an extensive application of the PCL–PEG blends in bone tissue engineering to mimic the extracellular matrix of the bone or to deliver osteogenic factors.^23^

Ideally, to boost the process of osteogenesis, synthetic bone substitutes should provide an osteoinductive environment alongside being structurally similar to the bone tissue.^24^ In this environment, bone cells are induced to differentiate into osteogenic phenotypes. This differentiation cannot be solely achieved by relying on the inherent composition of the scaffold.^25^ To address this issue, incorporating pro-osteogenic molecules [e.g., growth factors (GFs) of cytokines] has widely been explored as an approach to induce healing of the bone.^26^ One of the primary GFs used for bone tissue regeneration is the bone morphogenetic protein 2 (BMP-2).^27,28^ It shows outstanding pro-osteogenic behavior by inducing multipotent stem cells to differentiate toward the osteogenic lineage. The recombinant version of BMP-2 (rhBMP2) has been widely studied in the context of synthetic scaffolds for bone regeneration. However, these biomaterials frequently exhibit poor release kinetics that require the supraphysiological dosing of BMP-2, which has been linked to ectopic bone formation and cytotoxicity. These limitations are commonly being addressed using nanomedicine where the relevant GFs are encapsulated within nanoparticles to enhance spatiotemporal control of the local release.^29,30^

One category of such nanoparticles that are widely used in nanomedicine is dendritic silica mesoporous nanoparticles (DSMNs).^31,32^ The use of silica/titania nanostructures, such as DSTNs (dendritic silica/titania mesoporous nanoparticles), holds promise in bone tissue regeneration due to their ability to ensure sustained and controlled release of relevant molecules and facilitate cellular uptake.^33^ The TiO_2_ layer in these nanostructures contributes to the creation of an oxidative microenvironment,^34^ promoting osteogenic differentiation of bone cells, and acts as a nucleation site for the deposition of hydroxyapatite (HA) during the mineralization process.^35,36^

The rationale behind choosing silica/titania nanostructures in this study lies in their unique features. The mesoporous structure of silica allows for efficient drug loading and slow release, providing precise control over release kinetics crucial for targeted therapy.^37^ Furthermore, their biocompatibility ensures the safety of these nanostructures for biomedical applications. Additionally, the photocatalytic properties of titania enable the generation of reactive oxygen species (ROS), contributing to the creation of an oxidative microenvironment. This strategic combination of features enhances the versatility and adaptability of silica/titania nanostructures for drug delivery in bone tissue engineering.^38^

ROS play a pivotal role in bone tissue regeneration, acting as signaling molecules essential for cellular activities like proliferation, differentiation, and migration. In the context of bone regeneration, ROS can influence osteogenic differentiation and support the formation of a mineralized bone matrix.^39,40^ However, maintaining a delicate balance of ROS is crucial, as an imbalance leading to oxidative stress can have detrimental effects on bone cells and tissues, impairing the healing process.

Curcumin (CUR) is the major bioactive polyphenolic ingredient of turmeric, which presents a wide range of biological properties, including anti-inflammatory, antioxidant, antimicrobial, anticancer, neuroprotective, and cardioprotective activities.^41,42^ Several studies have demonstrated the benefits of CUR in treating bone disorders and diseases such as osteolysis, periodontitis, rheumatoid arthritis, and osteoporosis. Furthermore, CUR has been shown to modulate inflammatory responses, osteoimmune environment, and osteogenic–osteoclastogenic coupling.^43,44^ For instance, the CUR-coated 3D-printed tricalcium phosphate (TCP) ceramic enhanced the mineralized bone formation by approximately 15% in rat distal femoral defect compared to the TCP alone, while the HA-coated titanium implants containing CUR resulted in greater osteointegration, osteoid formation, and total bone formation.^45^ Although CUR exhibits the relevant osteogenic properties, it accounts for low aqueous solubility, low absorption, and low bioavailability in the human body. Therefore, local and targeted delivery using nanoparticles is preferred to overcome these limitations. As a primary aim of this study, we investigate the potential of modified DSTNs, either alone or incorporated within a 3D scaffold, to locally release osteogenic molecules, i.e., the recombinant CUR and BMP2. Our matrix is a 5% (w/w) PCL–PEG matrix, with previously optimized physicochemical and mechanical features for bone regeneration, and we use it as a platform for the delivery of CUR-BMP-2@DSTNs.

This delivery setup can be further enhanced using low-intensity pulsed ultrasound (LIPUS) therapy. In recent years, LIPUS therapy has emerged as an FDA-approved physiotherapeutic technique to accelerate bone fracture healing as well as delayed or nonunion defects.^46^ The technique provides noninvasive stimulation to cells through acoustic pressure waves and enhances biochemical processes occurring within the cells.^47^ Previous in vitro studies have demonstrated that LIPUS stimulation increased the osteogenic activity of osteoblastic,^48,49^ stem,^50^ and progenitor cells.^51^ For instance, LIPUS stimulation has been reported to enhance the production of molecules by bone cells that are relevant to bone metabolism, such as cyclooxygenase-2 (COX-2) and prostaglandin E2.^52^ LIPUS has been used to improve the efficacy of DSNPs for drug delivery by increasing the permeability of cell membranes, which allows for more uptake of the nanoparticles and greater release of the therapeutic agent.^53^ This technology can also be used to stimulate tissue regeneration and to improve the performance of tissue-engineering scaffolds, as shown in the graphical abstract. In this study, we examine the impact of ultrasound waves on the release of CUR and BMP2 from DSTNs/PCL–PEG scaffolds as well as their effects on the osteogenesis of mesenchymal stem cells (MSCs) by only changing the operating time.

**Scheme 1:**
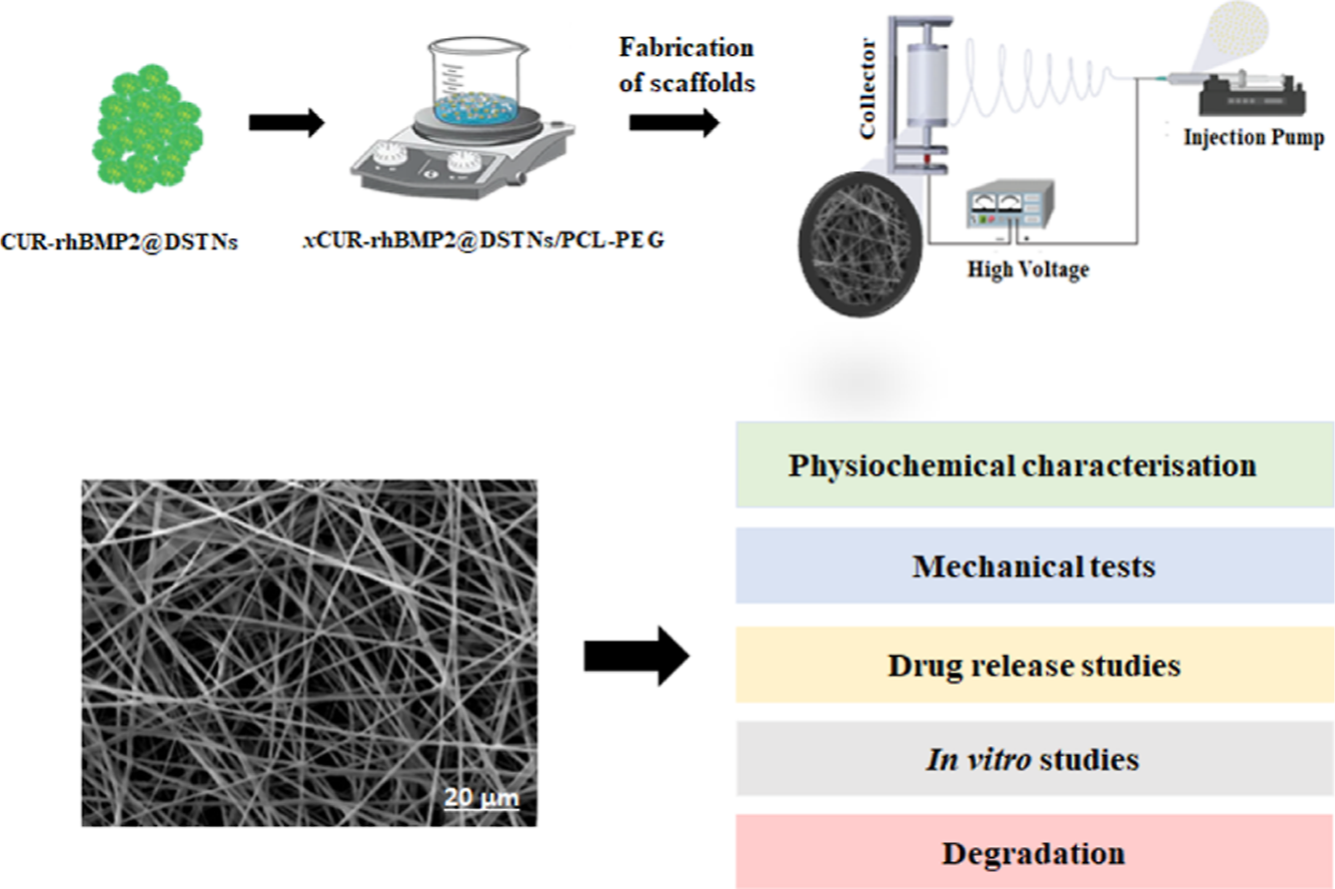
Summary of the Study (Study Design)

While it is true that other nanoparticles can possess similar features, in this study, we aim to develop a novel idea based on our previous work on these dendritic nanoparticles.^53^ This study delves into the potential of these particles as carriers for drug delivery in tissue engineering under ultrasonic (US) irradiation. Specifically, we focus on dendritic silica/titania mesoporous nanoparticles and aim to assess their capability for the controlled codelivery of CUR and BMP-2. Our goal is to enhance the osteogenesis of MSCs. To achieve this, we incorporated these nanoparticles into PCL–PEG at different weight percentages (0, 2, 5, 7, 9, and 10 wt %) to create distinct scaffolds labeled S0, S2, S5, S7, S9, and S10, respectively. These scaffolds underwent comprehensive evaluations, including analyses of release profiles and their potential to induce osteogenic differentiation of MSCs, with or without LIPUS treatment. Scheme 1 summarizes the key steps and studies conducted in this work.

## Materials and Methods

### Materials

Poly(caprolactone) (PCL, MW: 80,000), poly(ethylene glycol) (PEG, MW: 20,000), tetraethyl orthosilicate, 3-(trimethoxysilylpropyl) diethylenetriamine (Si-DETA), 3-(4,5-dimethylthiazol-2-yl)-2,5-diphenyl tetrazolium bromide (MTT), cetylpyridinium bromide, RPMI 1640, and CUR were purchased from Sigma-Aldrich. Tetrabutyl titanate [Ti(OBu)_4_], ammonium nitrate, *n*-butanol, cyclohexane, and other pure solvents were purchased from Merck. rhBMP-2 and alkaline phosphatase assay kit were purchased from Abcam (Cambridge, UK).

### General Measurements

FT-IR JASCO 680-PLUS and JASCO-570 UV–vis spectrometers were used to record FT-IR and UV–vis spectra, respectively. A Philips X-ray diffractometer using a nickel-filtered Cu Kα radiation (λ = 1.5418 Å) in the 2θ range = 10–100° was used to record X-ray diffraction (XRD) patterns. A JEOL scanning electron microscope was used to conduct field-emission scanning electron microscopy (FE-SEM) to determine the shape of the scaffolds. A conductivity meter (Jenway, model 3540, England) was used to measure the electrical conductivity (EC). Hydrophilicity was characterized by the water contact angle (WCA) test (ASTM D5946). Thermogravimetric analysis (TGA) with a Q50 TGA model from TA Instruments was used to assess the thermal stability of the copolymers. The pH of the solutions was measured by a combined electrode and Metrohm pH meter.

### Functionalization of DSMNs with a Titania Layer

First, DSMNs were synthesized and characterized according to our previous study. In the next step, dendritic silica/titania nanoparticles were synthesized based on our previous work.^53,54^ We added 250 mg of DSMNs to 180 mL of acetonitrile/ethanol (1:1, v/v %). After adding Ti(OBu)_4_ (0.7 mL) to 20 mL of acetonitrile/ethanol (1:1, v/v %), the produced mixture was stirred for 2 h at room temperature. The milky precipitate was centrifugated and dried in a vacuum at room temperature overnight.

### BMP-2 and CUR Loading into DSTNs Mesoporous Channels

The nanocarrier channels were loaded with CUR and BMP-2 using a solvent deposition procedure. The DSTNs (with a cargo to nanoparticle ratio of 1:2) were dispersed in a 25 mL EtOH solution of CUR. Then, the mixture was stirred at room temperature for 48 h. Centrifugation was used to collect the CUR@DSTNs. The final product was washed three times, and the remaining solvent evaporated at room temperature under vacuum conditions. In the next step, 0.4 mg/mL BMP-2 solution was prepared by dissolving the lyophilized BMP-2 in dilute 0.05 M acetic acid. Then, 0.6 g of CUR@DSTNs was added to 25 mL of prepared BMP-2 solution. The obtained solution was put in a refrigerator shaker with a speed of 80 rpm at 37 °C for 12 h. The suspension was centrifuged and then CUR-BMP-2@DSTNs was collected. The final drug delivery system contained a total of 45 wt % CUR and 89 wt % BMP-2.

### Preparing Solutions for Electrospinning

To prepare solutions for electrospinning, PCL–PEG (1:1 wt %) was dissolved in a methanol/chloroform solvent (9:1 v/v %), and then different concentrations of *x*CUR-BMP-2@DSTNs (*x* = 0, 2, 5, 7, 9, and 10 wt %) were added to the polymer solution and stirred vigorously overnight. The notation “S0” represents the incorporation of drugs into the pure (*x* = 0) PCL–PEG scaffold. The choice of weight percentages for loading drug and protein particles was determined through a diligent process that involved rigorous preliminary studies. These weight percentages were carefully selected to optimize both the biocompatibility and controlled release properties of the materials. The obtained suspensions were fed into a 1 mL syringe which has a blunted 23-gauge needle made of stainless steel. Uniform nanofibers were synthesized by adjusting electrospinning parameters as follows: temperature: 24–26 °C, relative humidity: 28–30%, voltage: 17–22 kV, gap distance: 10–15 cm, and collection time: 2 h. The concentration of the spinning solutions was 10 to 20% w/v with a flow rate of 2 mL/h. Aluminum foils were used to collect the electrospun scaffolds, which were named S0, S2, S5, S7, S9, and S10 (for example, S2 indicates the electrospun scaffold with 2 wt % CUR-BMP-2@DSTNs, S5 5 wt % CUR-BMP-2@DSTNs, and so on). After electrospinning, the scaffolds were collected on a substrate. To remove any residual solvent or moisture, the samples were placed in a controlled drying environment of a desiccator.

### Scaffold Characterization

The morphology of the electrospun scaffolds was analyzed using a TESCAN MIRA-3 scanning electron microscope after drying under vacuum and sputter-coating with gold–palladium. ImageJ software was used to determine the fiber diameter and porosity. XRD measurements, conducted at room temperature, employed a scanning speed of 4°/min and a chart speed of 20 mm/min, exploring the diffraction pattern from 10 to 100°. TGA assessed the thermal stability by subjecting samples to a 10 °C/min heating rate in a 60 mL/min argon flow, reaching 800 °C on a platinum pan. Uniaxial tensile testing of electrospun scaffolds utilized a MACH-1 mechanical tester, with samples cut into 50 mm × 10 mm rectangles and tested at a 0.5 mm/s velocity. Hydrophilicity was determined using a contact angle system, measuring the WCA at *t* = 0 s after dropping 3 μL of distilled water on the scaffold surface, and the surface wettability was calculated based on the average contact angle from three repetitions. Water uptake was assessed by the difference in weight between dried (*W*_dry_) and immersed (*W*_wet_) samples, using the following equation: water uptake (%) = (*W*_wet_ – *W*_dry_)/*W*_wet_ × 100. The soaked samples were weighed after removing residual surface water in 10 mL vials filled with distilled water.

### Biodegradation Analysis

The scaffolds were sterilized before analysis using UV irradiation and 70% (v/v) ethanol–distilled water for 1 h. The mechanical and structural properties of scaffolds were assessed in a simulated environment like in vivo by biodegradation analysis. The scaffolds were incubated in a lipase solution [110 U/L in phosphate-buffered saline (PBS)] in different periods ranging from 0 to 28 days (0, 7, 14, 21, and 28 days), daily refreshing the lipase solution. The scaffolds were washed, dried, and then weighed at the end of each period, and the percentage of weight loss was recorded.

### In Vitro Release of CUR and BMP-2

To place the nanofiber containing the CUR and BMP-2 delivery system into 48-well plate wells, they were cut into square shapes with a 10 mm side length and then placed in 0.5 mL of PBS (pH 7.4). Then, the wells were shaken in an incubator shaker at 37 °C with 100 rpm. The release of CUR and BMP-2 was checked on different days using a spectrophotometer. A fresh buffer solution was used to refresh solutions at each sample collection. To control the drug release, ultrasound pulses were applied with a source intensity of 1 W/cm^2^ and a frequency of 1.0 MHz. This frequency is selected based on its therapeutic effects and reasonable penetration in tissues, as reported in previous research or clinical reports.^52^ A duty cycle of 20% was set for the pulsation of ultrasound waves over 10 min on each day. The serial dilution of known CUR and BMP-2 concentration was used to generate the standard curve to estimate CUR and BMP-2 concentrations, and the mean and standard deviation of three replicates of each sample were calculated. To address the issue of protein extraction and accurately calculate the actual amount of BMP-2 released from carrier materials, a corrected method was employed. The BMP-2 concentration was determined using a BMP-2 ELISA kit.

### Cell Culture

The human osteosarcoma cell line (MG-63) was used for in vitro studies. The MG-63 cells were held at 37 °C in a humidified atmosphere of 5% CO_2_ using DMEM with 10% (v/v) fetal bovine serum. In the cell culture experiment, an osteogenic medium was created by supplementing DMEM + 10% FBS with ascorbic acid (25 μg/mL) and β-glycerophosphate. This modification aimed to enhance collagen synthesis, extracellular matrix mineralization, and alkaline phosphatase activity, promoting osteogenic differentiation in the cultured cells. The scaffolds were sterilized for 4 h with 70% ethanol. The scaffolds were washed with PBS solution for 30 min and were gently shaken. This process was repeated three times before cell seeding. Then, 100 μL of cell suspension was seeded at a seeding density of 1 × 10^4^ cells/well/scaffold in a 24-well plate over the scaffolds. Then, the seeded scaffolds were kept for 2 h at 37 °C in a humidified atmosphere of 5% CO_2_. Eventually, 1000 μL of culture media was added to cover the scaffold surface and was replaced every 48 h.

### Cell Viability and Proliferation Assessment

The viability and proliferation of cultured cells on the scaffold were assessed using the MTT assay. After punching the samples and putting them at the bottom of the 96-well plate, the samples were sterilized for 15 min using UV light. Then, MG-63 cells were seeded at 7 × 10^3^ cells/sample density and were incubated for 1 to 3 days under standard culturing conditions. Whenever the culture media was depleted, the cells were washed using PBS three times, and then 150 μL of new DMEM containing 20 μL of 5 mg/mL MTT solution was added to each well and incubated for 4 h. As soon as the purple formazan crystals were formed, the media was replaced with 100 μL DMSO for dissolving the formazan crystals. Finally, the 100 μL aliquots were moved to a 96-well plate containing three replicates per sample, and a microplate was used to read the absorption at 570 nm.

### Alkaline Phosphatase Activity

Alkaline phosphatase (ALP) activity on cell-seeded nanofibers was evaluated using an alkaline phosphatase kit. This allowed us to quantify ALP activity on the nanofiber scaffolds seeded with MG-63 cells, which is an important indicator of the differentiation and mineralization potential of cells. Briefly, MG-63 cells were seeded on the nanofiber scaffolds at a density of 4 × 10^4^ cells/well in 96-well plates. After the culture period, the enzyme–substrate solution containing *p*-nitrophenyl phosphate (pNPP) was added to and incubated for 60 min at room temperature. To end the reaction, the Stop solution was added, and to calculate the activity of ALP on the cell-seeded nanofiber scaffolds the amount of released *p*-nitrophenol was measured at 405 nm. The higher the ALP activity, the more the release of *p*-nitrophenol, and therefore the higher the absorbance at 405 nm.

### Statistical Analysis

In the present study, data analysis was conducted employing SPSS Statistics software, version 20. To assess the differences in mean quantities among the study groups, a one-way analysis of variance was performed. Each data point was replicated three times, and standard deviations were calculated using the formula for standard deviation as the square root of variance, where the variance is the average of squared differences from the mean. Statistical significance was defined as a *P*-value less than 0.05 (*P* < 0.05).

## Results and Discussion

### Synthesis and Structural Characterization

**Figure 1:**
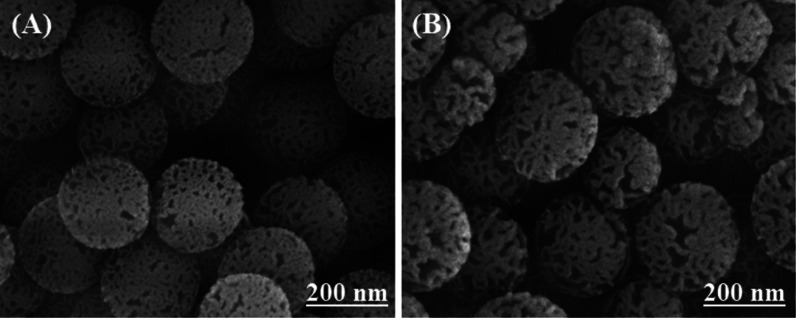
SEM images illustrating the powder morphology of (A) dendritic silica mesoporous nanoparticles (DSMNs) with PDI = 0.04 and (B) dendritic silica/titania mesoporous nanoparticles (DSTNs) with PDI = 0.02.

The morphological characteristics of dendritic silica nanoparticles (DSMNs) were examined through SEM both before and after the introduction of TiO_2_ nanoparticle coating, as illustrated in Figure 1. Postcoating, the particle size analysis conducted via DLS revealed a size transition for DSMNs, shifting from an initial mean size of 210 ± 6.1 to 221 ± 11 nm for DSTNs. The polydispersity index (PDI), indicative of particle size distribution uniformity, consistently maintained low, measuring 0.04 for DSMNs and 0.02 for DSTNs. These narrow particle size distributions provide additional insights into the precision of the coating process. We determine standard deviation by assessing variation among three replicates.

**Figure 2:**
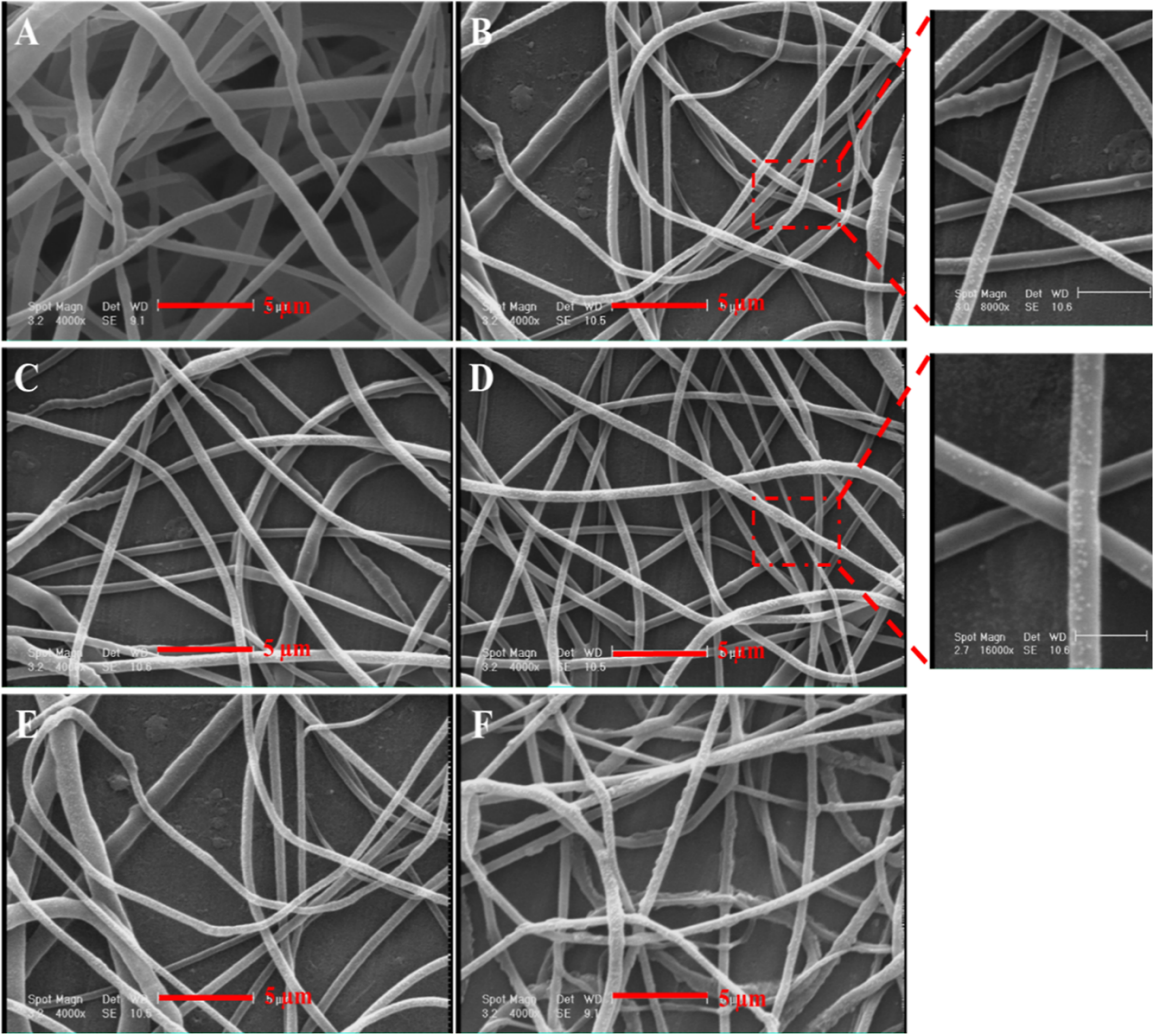
SEM images of nanofibers with different nanoparticle concentrations (0, 2, 5, 7, 9, and 10 wt %) labeled (A) S0, (B) S2, (C) S5, (D) S7, (E) S9, and (F) S10, respectively.

In Figure 2, FE-SEM images of electrospun scaffolds are presented. The findings reveal that the nanofibers of PCL/PEG (S0) exhibited irregular sizes with a broad size distribution and lacked uniform morphology. However, the introduction of DSTNs resulted in the formation of more consistent nanofibers with a narrower size distribution. Notably, this incorporation of nanoparticles led to a reduction in the average nanofiber diameter, as detailed in Table 1. These observations can be attributed to the increased EC of the polymeric solution achieved by the addition of DSTNs.

**Table 1:** Measured Characteristics of Nanocomposites (Each Measurement Was Conducted Three Times and the Numbers Include Standard Deviation)^a^

sample no.	nanofiber diameter (nm)	ave. porosity (%)	ave. water contact angle (deg)	ave. water absorption (%)	electrical conductivity (S cm^–1^)
S0	470 ± 87	69.34 ± 1.4	125 ± 10	15	7.12 ± 1.34
S2	452 ± 56	67.51 ± 2.1	103 ± 45	28	9.35 ± 1.41
S5	431 ± 45	64.71 ± 1.6	97 ± 18	32	12.57 ± 1.34
S7	389 ± 73	61.03 ± 2.6	85 ± 32	44	14.66 ± 1.45
S9	367 ± 89	58.67 ± 0.9	73 ± 22	51	17.34 ± 1.27
S10	356 ± 63	49.89 ± 1.7	65 ± 34	55	21.64 ± 1.12

a S*i* (*i* = 0, 2, 5, 7, 9, 10) stands for the electrospun scaffold with *i* wt % CUR-BMP-2@DSTNs.

Furthermore, it was noted that when high concentrations of DSTNs were added (S9 and S10), adverse effects on the nanofiber morphology were observed, as seen in Figure 2 (panels E and F). These structural deformities in S9 and S10 can be attributed to the detrimental impact of the high nanoparticle concentration on the integrity of the polymer chains and their stretching during the electrospinning process. The higher concentration of surface charges facilitated the attachment of more nanoparticles to the fiber surfaces as agglomerates, resulting in a rougher surface texture.^23^

Porosity is an important property of scaffolds, influencing cell infiltration, nutrient/waste exchange, and tissue regeneration uniformity. We measured scaffold porosity, shown in Table 1, and found that the inclusion of DSTNs reduced the porosity from 69.34 ± 1.4% in S0 to 61.03 ± 2.2% in S7. We utilized a multistep approach to measure scaffold porosity. First, we employed SEM to capture detailed images of the electrospun scaffolds. Subsequently, we utilized MATLAB for image processing and analysis. The process involved image preprocessing, thresholding, and particle analysis to identify and quantify the pores within the scaffold structure. The porosity was then calculated as the ratio of the total pore area to the total image area. This integrated SEM and MATLAB methodology provided us with a robust and quantitative measurement of scaffold porosity. The reduction in porosity from S0 to S7 is attributed to the decrease in nanofiber diameter, from 470 ± 87 nm (S0) to 389 ± 73 nm (S7). When the diameter of the nanofibers decreased from 470 ± 87 nm (S0) to 389 ± 73 nm (S7), the fibers were packed more tightly. Fibers with a smaller diameter occupied less space; this led to fewer gaps between them, thereby reducing the overall porosity. Even though more nanoparticles might be present, the reduction in fiber diameter outweighed the effect of increased nanoparticle concentration, resulting in decreased porosity.^55^

The diameter was calculated based on SEM images using ImageJ software. Additionally, we found that the initial EC of the PCL/PEG solution was 7.12 ± 1.34 S cm^–1^ but remarkably increased to 14.66 ± 1.45 S cm^–1^ for S7 nanofibers. The addition of silica nanoparticles to a PCL–PEG scaffold enhanced the electron conductivity by providing conductive pathways and facilitating efficient charge transfer. The nanoparticles improved interfacial contacts within the polymer matrix, creating synergistic effects that contribute to a higher overall electron conductivity in the composite material. This enhanced conductivity highlights the potential of the scaffold to replicate and amplify natural electrical cues essential for bone tissue regeneration.

Scaffold wettability is a critical factor influencing biomolecule and cell interactions, directly impacting the regenerative potential of the scaffold. Hydrophobic surfaces can induce protein adsorption through hydrophobic interactions, potentially leading to conformational changes and immunological responses against scaffolds.^56^ In contrast, hydrophilic surfaces promote the preservation of native protein structures.^57^ Therefore, adjusting surface wettability is pivotal for enhancing scaffold regenerative efficacy. The incorporation of DSTNs onto PCL/PEG nanofibers significantly increased hydrophilicity, as evidenced by reduced water contact angles, see Table 1. This enhanced hydrophilicity facilitated greater water absorption capacity, fostering a moist microenvironment vital for cell attachment, proliferation, and tissue regeneration. Table 1 demonstrates that pure PCL/PEG nanofibers displayed a WCA of 125° ± 10°, indicating their hydrophobic nature. However, the incorporation of DSTNs onto the nanofibers significantly reduced the WCA, rendering them more hydrophilic. This phenomenon is attributed to the inherent hydrophilic nature of silica-based materials. The presence of hydroxyl groups on the surface of DSTNs promotes interactions with water molecules, reducing the surface tension and making the nanofibers more prone to wetting, resulting in a lower WCA.^58^

**Figure 3:**
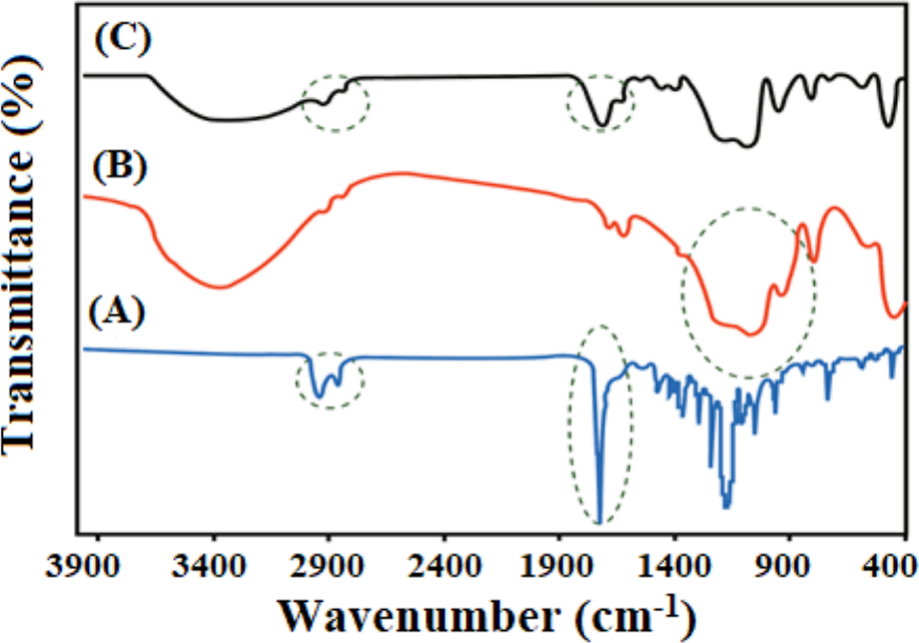
FTIR spectra of (A) S0 (pure PCL/PEG blend), (B) DSTNs, and (C) S7 (7 wt % CUR-rhBMP-2 nanoparticle concentration).

We also investigated surface functional groups and chemical interactions within the nanocomposite using FTIR spectroscopy, and the resulting FTIR spectra for various samples are depicted in Figure 3. In the FTIR spectrum of DSTNs, distinct peaks were identified at 463, 804, and 1100 cm^–1^, signifying the bending, symmetric stretching, and asymmetric stretching vibrations of Si–O–Si bonds, respectively. These observations align with earlier studies and affirm the presence of intended silica-based functional groups on DSTNs’ surface. The FTIR spectrum of PCL/PEG displayed characteristic peaks at 1724 and 1240 cm^–1^, attributed to the C=O stretching and C–O–C stretching vibrations in the PCL component of the nanocomposite. The FTIR spectrum of the nanocomposite featured peaks corresponding to both DSTNs and PCL/PEG components, serving as evidence for the successful formation of the nanocomposite. This confirms the presence of the desired functional groups and chemical interactions between the components.^3,19^

**Figure 4:**
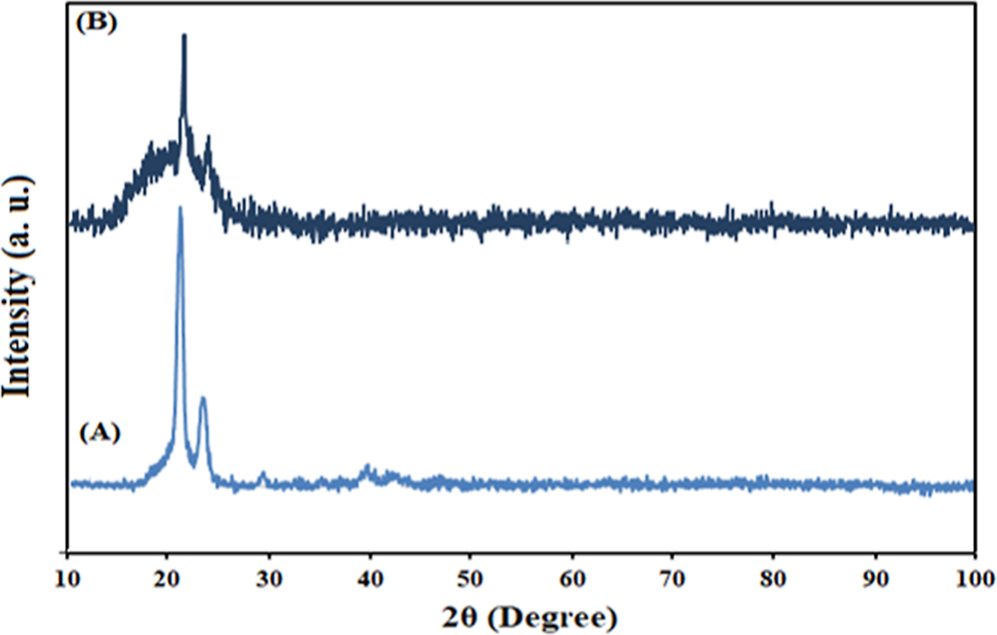
XRD patterns for (A) S0 (pure PCL–PEG) and (B) S7 (7 wt % nanoparticle concentration) scaffolds.

XRD patterns provide valuable insights into the crystalline nature of the samples. XRD analysis was performed on the prepared nanocomposites, and the patterns are presented in Figure 4. In XRD pattern A, which is for sample S0, two prominent diffraction peaks are observed at Bragg angles of 2θ = 23.6 and 21.3°. These peaks are attributed to the (110) and (200) crystallographic planes, respectively, of the orthorhombic crystal structure of PCL/PEG. These peaks indicate the presence of a well-defined crystalline structure in the PCL/PEG component of the nanocomposite. In contrast, XRD pattern B, which is for sample S7, exhibits a broad peak at 2θ = 22.8°, which suggests the amorphous nature of the DSTNs. The broad peak indicates a lack of long-range order, typically associated with the crystalline materials. Based on these observations, it can be concluded that the nanoparticles have been successfully incorporated into the PCL/PEG copolymer structure.^3^

**Figure 5:**
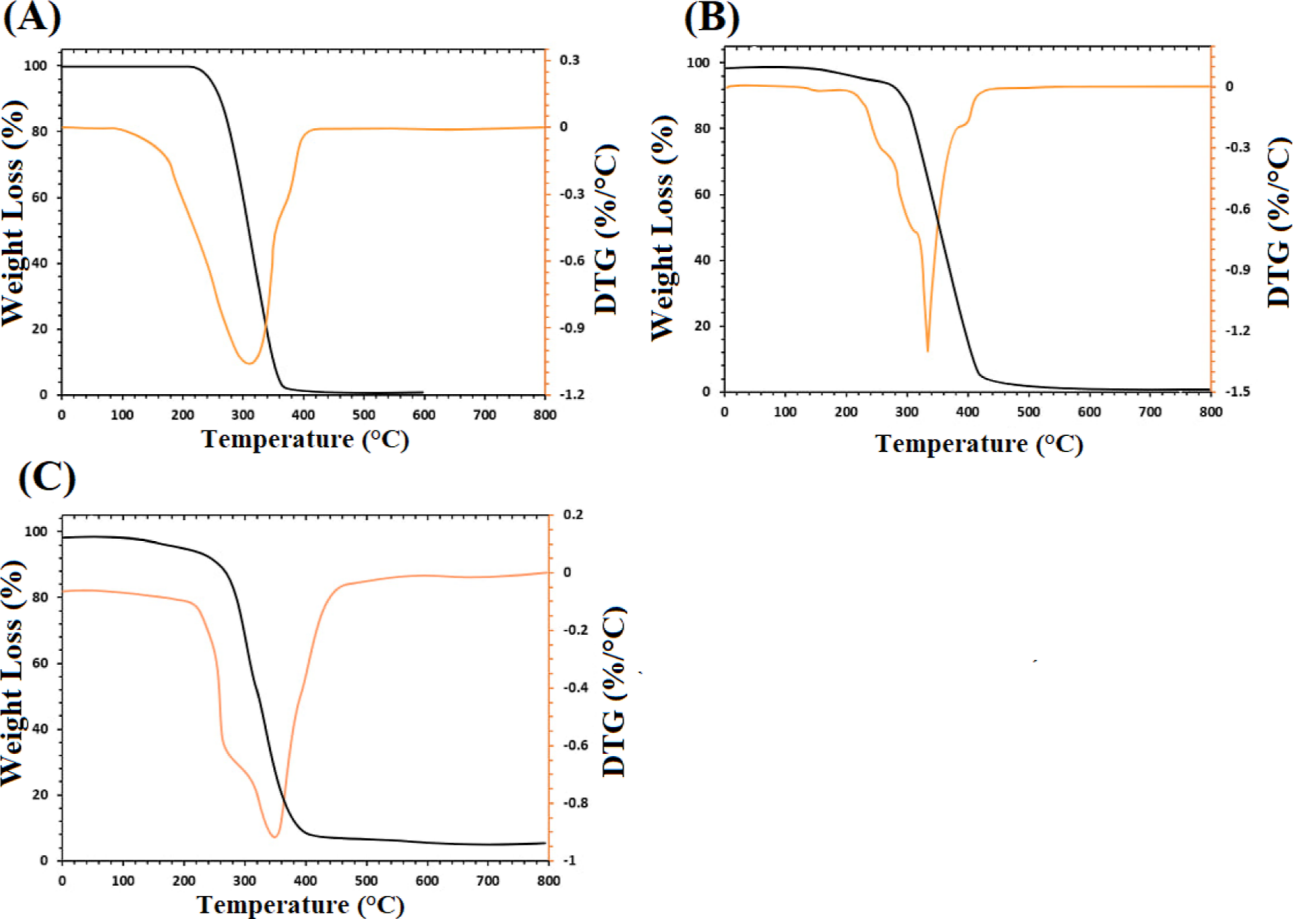
TGA and DTG curves for (A) S0 (pure PCL–PEG), (B) S2 (2 wt % nanoparticle concentration), and (C) S7 (7 wt % nanoparticle concentration) scaffolds.

The thermal stability of a material primarily depends on its structure and the bonds that hold this structure together. Thermal stability and decomposition kinetics of the prepared scaffolds were evaluated using the TGA technique, and results are presented in Figure 5. Before the temperature reaches 300 °C, a slight weight loss is observed, which can be attributed to the removal of physically adsorbed water from the surface of all scaffolds. This initial weight loss is evident in the TGA weight loss curve. The onset temperature for the decomposition of the scaffolds is around 300 °C, signifying the point at which the scaffolds begin to degrade. This information is visible in the TGA weight loss curve.

As demonstrated in Figure 5, the incorporation of DSTNs has a positive impact on the thermal stability of the nanocomposites. This is supported by the DTG curve, which shows the rate of weight loss as a function of temperature. Among the samples, S2 exhibits the least thermal stability as it starts to degrade at a lower temperature compared to S0 and S7 scaffolds. In the DTG curve, this lower initiation temperature is depicted. Conversely, an improvement in the thermal stability of the S7 scaffolds is observed, which is due to the strong interaction between the polymer and the DSTNs.

**Figure 6:**
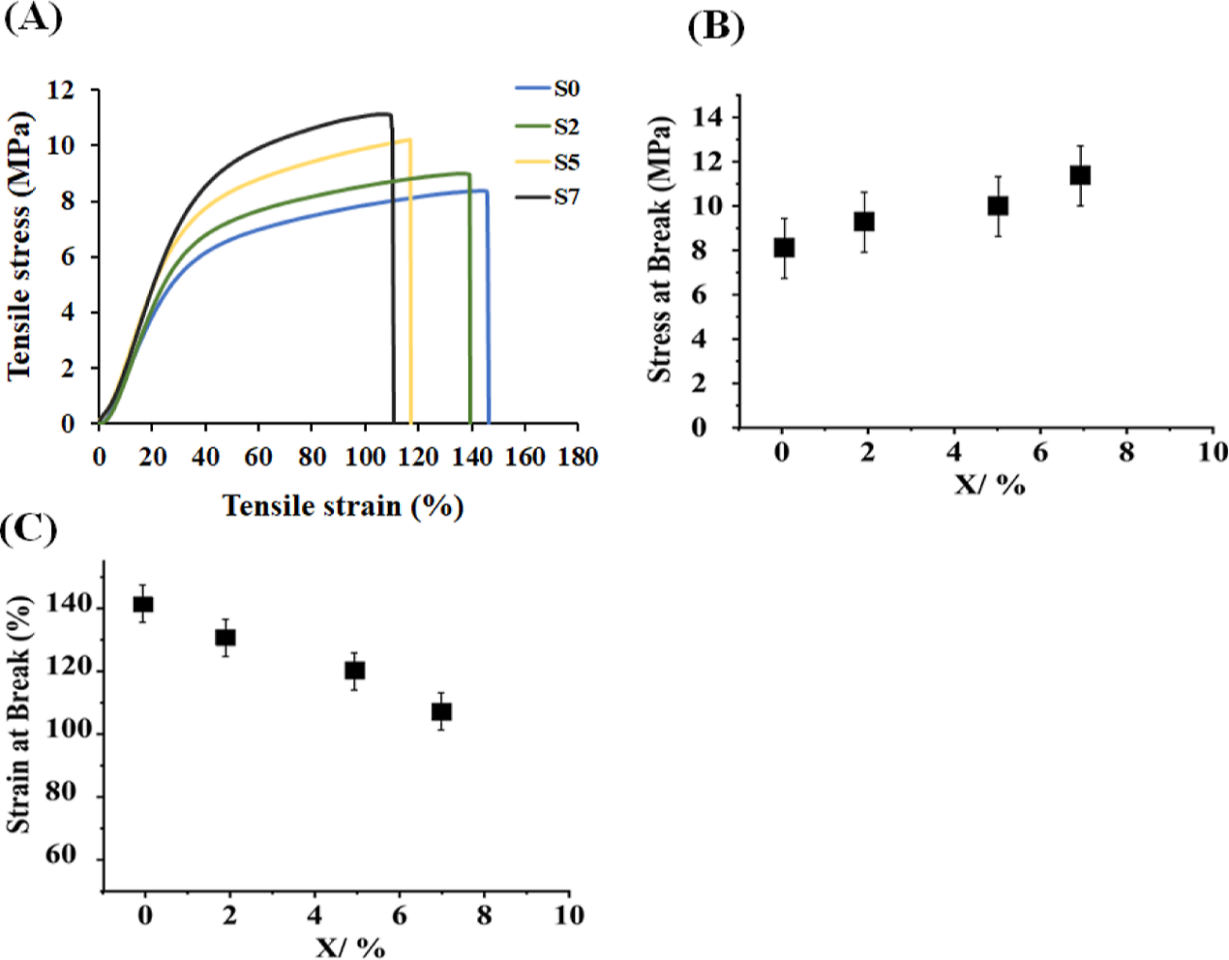
Results of tensile stress tests. (A) Stress–strain curves and (B,C) ultimate stress and maximum strain at the breaking point vs nanoparticle concentration. Nanofibers with different nanoparticle concentrations (0, 2, 5, and 7 wt %) are labeled S0, S2, S5, and S7, respectively. The error bars represent standard deviation.

The tensile behavior of the electrospun samples was evaluated and the related stress–strain curves are shown in Figure 6. The tensile strength (ultimate normal stress that the sample can hold before it breaks) of the nanofiber sample labeled S0 was approximately 8.0 MPa. By increasing the concentration of CUR-BMP-2@DSTNs, the tensile strength gradually increased to ca. 9.0 MPa for S2, 10.0 MPa for S5, and 11.0 MPa for S7 nanofibers (Figure 6B). Although not distinguishable on the curve, the Young’s modulus also increased upon adding more CUR-BMP-2@DSTNs to the samples. The increase in the tensile strength and Young’s modulus indicated that the structure became stronger against normal stresses—a fact that is crucial for bone tissue regeneration.^4^ Moreover, the normal strain (percentage of change in the sample length) at break for all samples ranged from 145% for S0 to 110% for S7. Although the inclusion of more DSTNs led to an increase in the tensile strength, it resulted in a smaller elongation of the samples before their break. Notably, among all the samples, the nanofiber membrane containing CUR-BMP-2@DSTNs at a concentration of 7% (sample S7) demonstrated the highest tensile strength of 11.0 MPa, while it stretched less than the other samples before it broke. It seems that adding more CUR-BMP-2@DSTNs leads to the manufacturing of a stronger nanofiber membrane with higher Young’s modulus and ultimate stress but more brittle fibers. The change in these mechanical properties of the samples is mainly due to the change in their structure and not surface properties. The former can be seen in the XRD analysis where we found that adding more CUR-BMP-2@DSTNs leads to a more amorphous nature of the nanofibers. The broad peak we observed in Figure 4B indicates that the internal structure of the nanofiber shifts from a well-structured crystalline to a more amorphous one. Therefore, the structure becomes stronger against normal stress, and at the same time it cannot stretch as before due to its amorphous nature and hence becomes more brittle. More in-depth analysis on the matter is left for further studies; however, our mechanical testing results reinforce the significance of appropriate material selection and fabrication techniques for bone tissue regeneration.

## Results of In Vitro Studies

### Biodegradation

**Figure 7:**
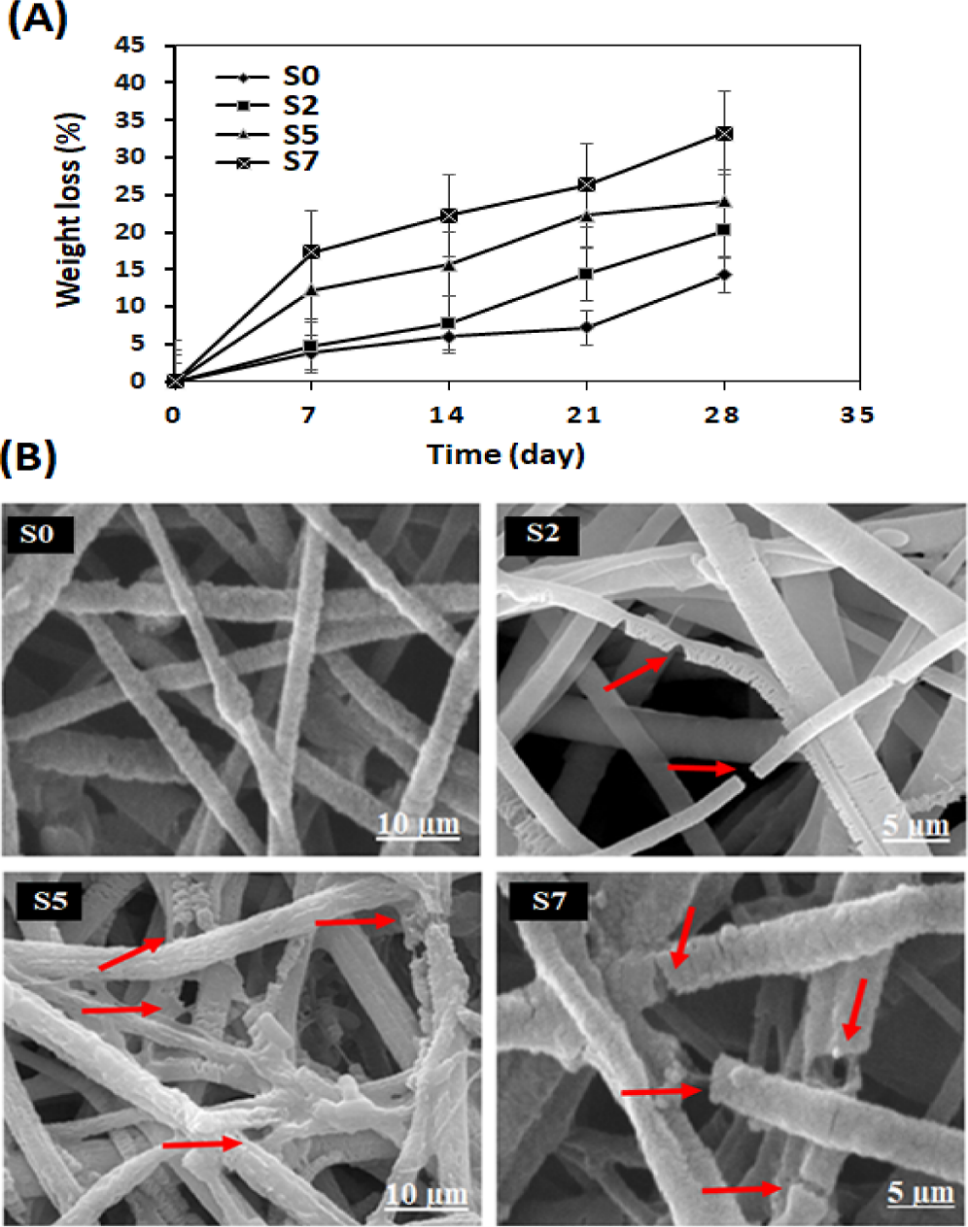
Degradation test results: (A) weight loss of scaffolds with different nanoparticle concentrations (0, 2, 5, and 7 wt % labeled S0, S2, S5, and S7, respectively) over 28 days and (B) corresponding SEM images of nanocomposite scaffolds after 28 days.

We quantified the weight loss and changes in the microstructure of the scaffolds using the gravimetric method and SEM imaging. As shown in Figure 7A, the pure PCL/PEG nanofibers (S0) showed the lowest biodegradation, less than 15% after 28 days. Incorporation of DSTNs accelerated the biodegradation of the scaffolds, and the highest biodegradation (ca. 35%) was observed in S7 scaffolds, which contain the highest amount of DSTNs. Moreover, the structural deformation of the scaffolds during the incubation time is indicated by red arrows in Figure 7B. It was observed that some of the nanofibers broke, and dissociation increased with the increase of the amount of DSTNs. The accelerated biodegradation upon increasing the amount of DSTNs is related to the effects of nanoparticles on the wettability and water absorption properties of the scaffolds.^59,60^ Hydrolysis is one of the main mechanisms involved in the biodegradation process, which strongly depends on the hydrophilic nature of scaffolds. Increasing the hydrophilicity of S7 accelerates the water diffusion and subsequently the degradation process with ∼35% weight loss in 28 days.

### Osteoactivity Findings

**Figure 8:**
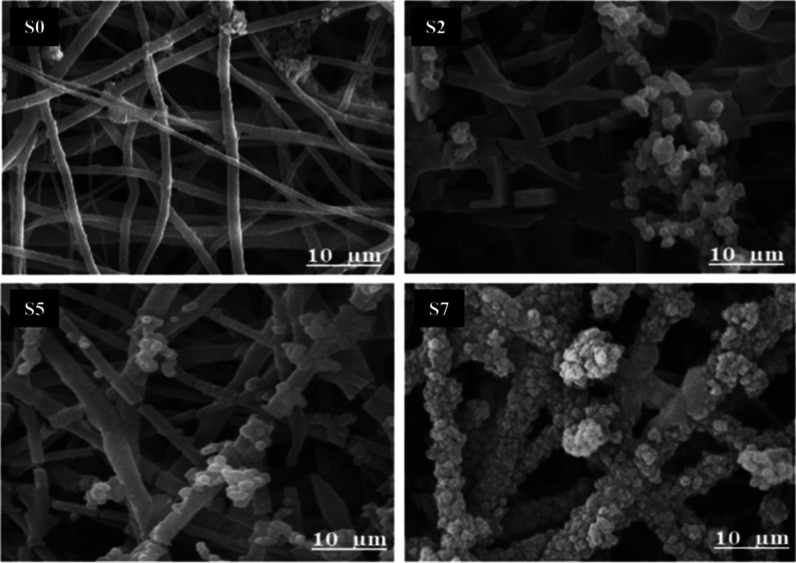
SEM images illustrating the mineralization process in SBF solution of nanocomposite scaffolds with different nanoparticle concentrations (0, 2, 5, and 7 wt % labeled S0, S2, S5, and S7, respectively) after 28 days.

The formation of calcium/phosphate ceramics on scaffolds in simulated body fluid (SBF) solution is considered the bioactivity of scaffolds dedicated to bone regeneration applications. We evaluated the interactivity of the prepared scaffolds in SBF solution for 28 days under ultrasound irradiation. The results showed that higher DSTNs content leads to the formation of more ceramics (more mineralization) on the nanofibers, see Figure 8. As shown in this figure, the S0 scaffold contains the lowest ceramic level, while in the S7 scaffold, the nanofibers are completely covered by calcium/phosphate ceramics, predominantly HA.^61^ This shows that one can expect the lowest bioactivity from the S0 (PCL/PEG) scaffold and the highest one from the S7. It can be attributed to the hydrophilic nature of DSTNs-containing scaffolds. Moreover, the DSTNs can act as nucleation sites for calcium/phosphate deposition and ceramic formation. More crevices on the surface of the scaffold created by the DSTNs lead to lower surface energy required for the physical deposition process. Moreover, ultrasound irradiation enhances the deposition process in a few ways. First, it improves the mixing, both in micro- and macroscales. This usually happens when one uses ultrasound irradiation at such relatively high frequencies. At 1 MHz, the viscous dissipation of the ultrasound wave is significant enough to enhance mixing, both in the bulk of the solution and close to the surface of the solid boundaries (acoustic streaming). Second, the mixing aids the growth of the calcium/phosphate crystals by improving the mass transport process via forced convection. Third, ultrasound can prevent agglomeration by mechanically shaking those agglomerates and lead to a more uniform distribution of the deposited ceramics on the surface of the scaffolds. Fourth, while the number of cavitation events at 1 MHz is reduced significantly compared to lower frequencies that are widely used to enhance sonochemical activities (e.g., 20 kHz), using high power levels, we still expect the formation of free radicals.^62,63^ Finally, similar to photovoltaic activity, the thermal dissipation of the ultrasound waves activates the TiO_2_ coating to generate ROS.^34^ These radicals assist the chemical deposition of ceramics on the surface of the fibers. Eventually, a more uniform, well-defined, and bioactive ceramic layer is deposited, which can be seen in Figure 8 for S7.

### Drug Release

**Figure 9:**
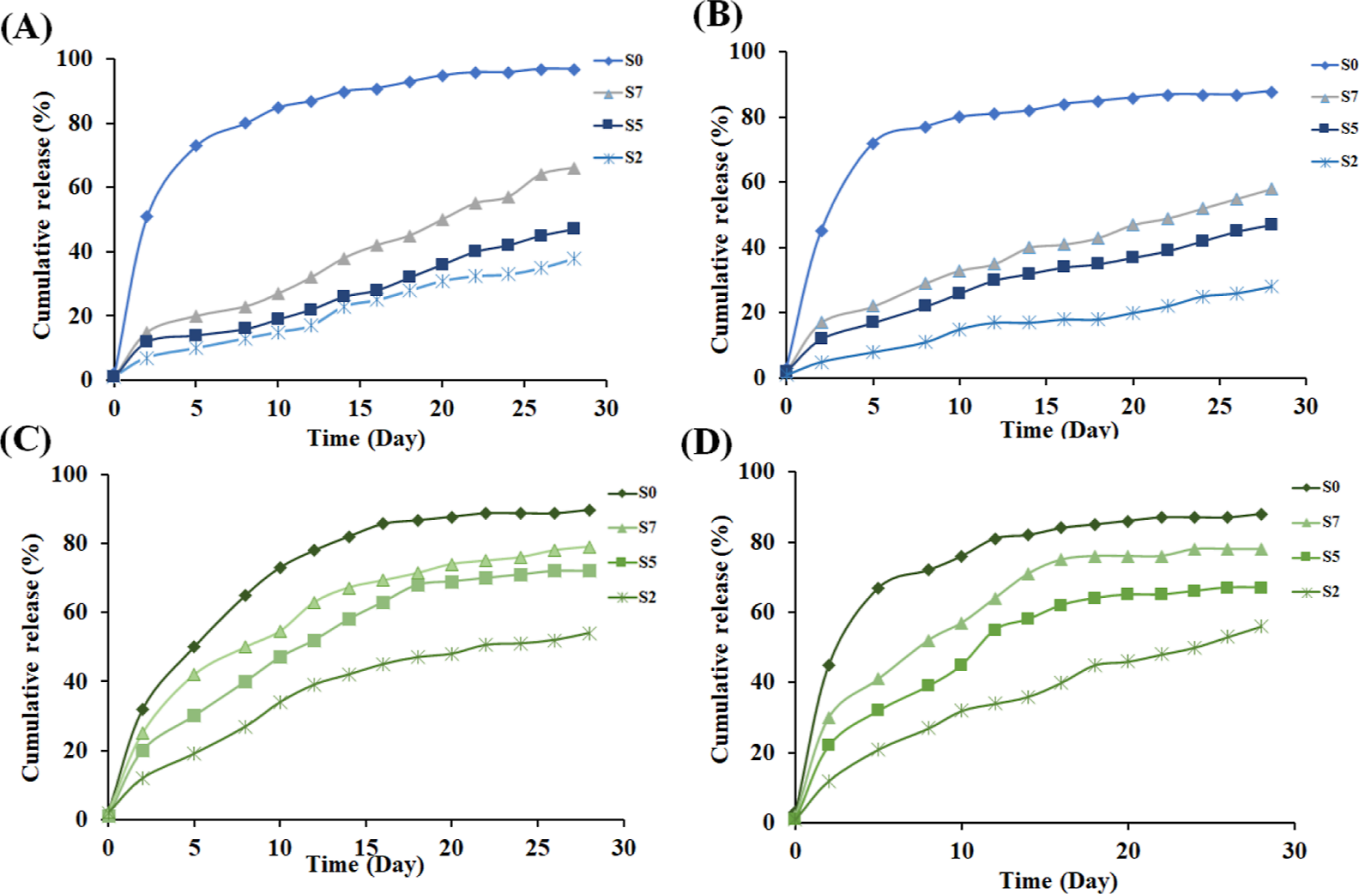
Drug release from the scaffolds in 28 days: release of CUR (A) and BMP-2 (B) without ultrasound irradiation; CUR (C) and BMP-2 (D) with ultrasound irradiation.

As stated before, in this study, two osteogenic agents, CUR with known osteogenic regulatory effects and BMP-2, a potent osteoinductive cytokine, were utilized. Figure 9 shows the drug release profiles of these agents, with and without ultrasound radiation over 28 days in SBF (pH = 7.6). The results show that approximately 90% of the CUR loaded into the scaffold S0 is released within 15 days, irrespective of using ultrasound irradiation. This is considered as a fast release, and the pattern indicates a lack of sustainability and control in the drug release process, highlighting the urgent need for an effective drug release system.

As shown in all panels in Figure 9, more sustained release was observed in the CUR-BMP-2@DSTNs scaffold with a higher concentration of nanoparticles (S7) compared to those with lower concentrations. Approximately 60% of the CUR and BMP-2 were released from the scaffold S7 without the use of ultrasonic irradiation. It is worth mentioning that the release of CUR from all scaffolds was higher than BMP-2, likely due to weaker interactions between this agent and the mesoporous channels during the drug loading process. In contrast, strong hydrogen bonds existed between BMP-2 and the carrier material. These findings highlight the consistent and significantly slowed release of both CUR and BMP-2.

Furthermore, apart from the local drug release through the scaffold, the study explored the potential for on-demand drug release using external triggers to enhance the effectiveness of the bone regeneration strategy. Ultrasound was applied as the external stimulus to trigger drug release. As shown in Figure 9, more sustained and controlled release was obtained under ultrasound irradiation, almost increasing the release by ca. 20% for all scaffolds. Using ultrasound allows for controlled and efficient induction of osteogenic effects through the continuous and controlled release of CUR and BMP-2, holding promise for advanced bone regeneration applications.^64^

### Cell Viability and Proliferation

**Figure 10:**
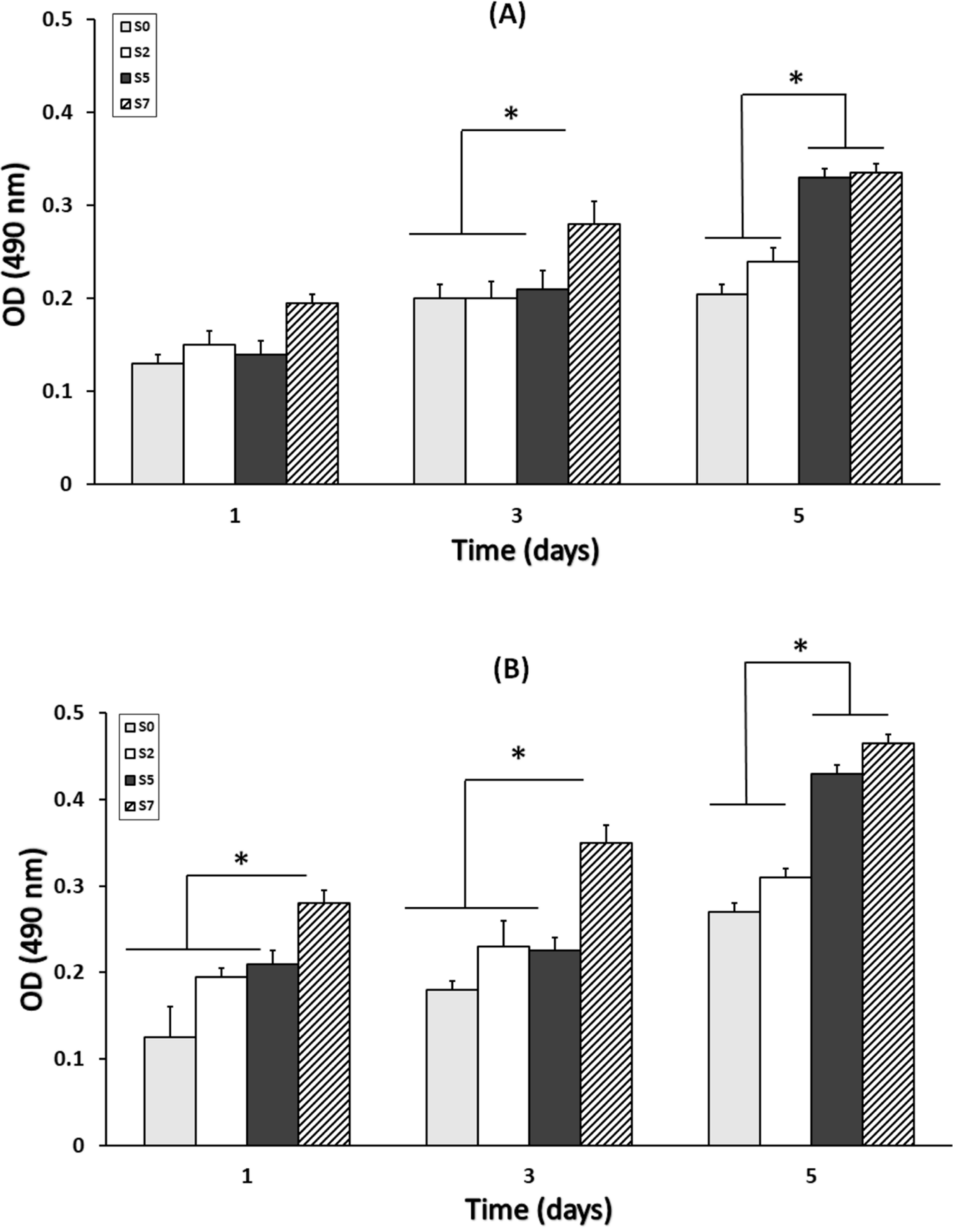
MTT assay results to evaluate the cell toxicity of four types of electrospun scaffolds on MG-63 cells at different incubation times (A) without and (B) with ultrasound irradiation (*n* = 3, *p < 0.05). Error bars represent standard deviation.

MG-63 cell viability and proliferation on the prepared scaffolds were measured using an MTT assay kit, and the results are presented in Figure 10. Proliferation percentage refers to the extent of cellular growth or reproduction on the respective scaffolds, quantified as a percentage increase in cell numbers over time. The results indicate that cell proliferation on scaffolds containing DSTNs was notably higher than that on pure PCL/PEG nanofibers at each observed time point. Moreover, the proliferation percentage for S5 and S7 was found to be significantly elevated compared to S0 and S2 (*p* < 0.05). The observed higher proliferation of cells on S5 and S7 can be attributed to their more hydrophilic nature, which promotes cell attachment, as well as higher amount of loaded drugs. Moreover, ultrasound stimulation had a substantial impact on cell behavior. In the presence of ultrasound, cell proliferation on all scaffold types was accelerated, surpassing the proliferation levels observed without ultrasound stimulation. Ultrasound acted as an external stimulus, effectively promoting cell growth and scaffold-mediated tissue regeneration.

A few hypotheses, although not generally accepted, such as mechanical stimulus that may lead to vigorous vibration of the scaffolds as well as change in the collagen deposition are reported in the literature to explain such improvement. The dual enhancement strategy, combining DSTNs within scaffolds and utilizing ultrasound stimulation, synergistically improved cell viability and proliferation, making the S7 scaffold particularly well suited for dedicated applications in bone regeneration. These findings underscore the potential of these scaffolds and ultrasound as a powerful combination for advancing bone tissue engineering and regenerative medicine.^49,52^

### Cells Morphology on Nanofibers

**Figure 11:**
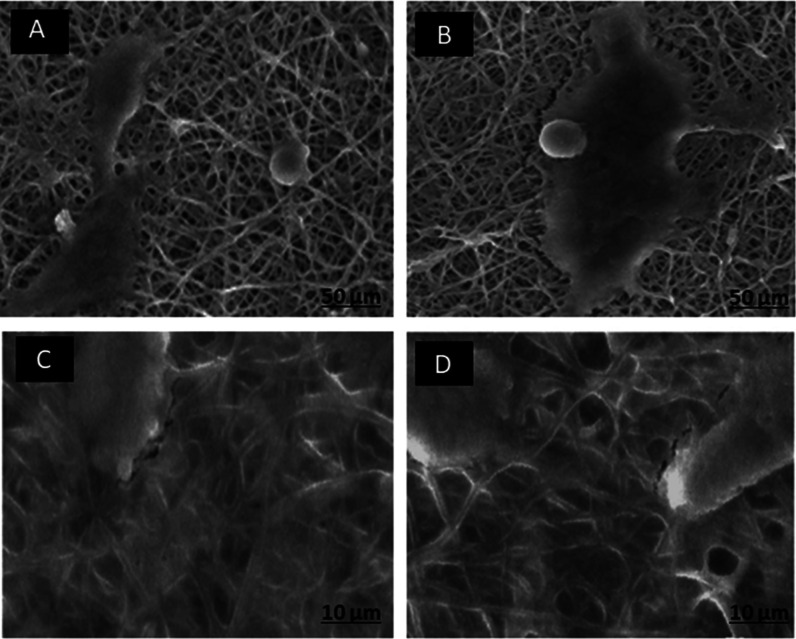
Morphology of MG-63 cells on the fabricated scaffolds (A) S0, (B) S2, (C) S5, and (D) S7 with different nanoparticle concentrations (0, 2, 5, and 7 wt %, respectively).

SEM analysis was conducted to investigate the morphology of MG-63 cells upon cultivation on the prepared scaffolds, as illustrated in Figure 11. The results reveal a significant difference in cell attachment patterns among the various scaffold types. Notably, MG-63 cells exhibited a higher degree of successful attachment to the surfaces of S5 and S7 nanofibers when compared to S2 and the control sample (S0). The enhanced cell attachment observed on S5 and S7 scaffolds can be attributed to their distinctive feature of well-incorporating DSTNs into the scaffolds. DSTNs serve a dual purpose in this context. First, they enhance the scaffold hydrophilicity, making it more water-friendly. This increased hydrophilicity can create a more inviting environment for cells to adhere to the scaffold surface.

Second, the DSTNs facilitate the controlled release of osteogenic factors such as CUR and BMP-2. These factors are known to promote the growth and differentiation of osteogenic (bone-forming) cells. Essentially, the presence of bioactive molecules like CUR and BMP-2, combined with the scaffold-improved hydrophilicity, provides an ideal setting for the cells to attach, grow, and ultimately contribute to the process of bone formation, which is important in various tissue engineering and regenerative medicine applications. Additionally, the increased surface area and nanoscale features of S5 and S7 nanofibers, owing to their smaller diameter, provide more anchoring sites for cell membrane receptors. Consequently, these promoted stronger cell–scaffold interactions lead to improved cell attachment and spreading. Similar behavior has been reported by Lee et al. using HA treated with citric acid, which significantly improved the physicochemical properties of HA, including the surface charge, leading to increased protein loading capacity, higher affinity for lysozymes and BMP-2, enhanced protein adsorption and sustained release, and nontoxicity that promoted osteoblast-like cell proliferation.^65,66^

### Alkaline Phosphatase Activity Results

**Figure 12:**
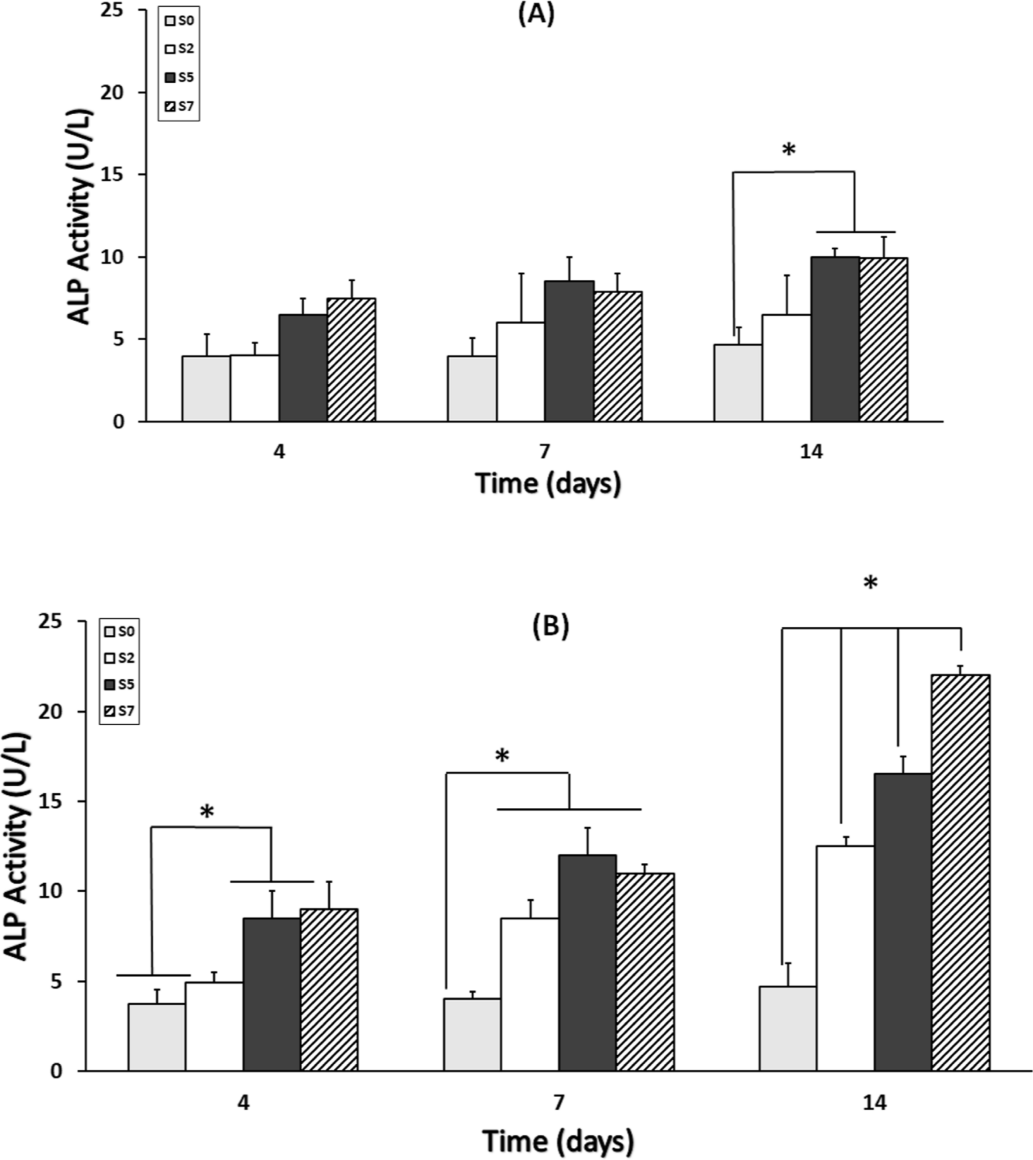
Alkaline phosphatase (ALP) activity test results for nanofibers: (A) without and (B) with ultrasound irradiation on days 4, 7, and 14 (*n* = 3, *p < 0.05). Error bars represent standard deviation.

The ALP activity in nanofiber scaffolds seeded with MG-63 cells provides crucial insights into the osteogenic potential and cellular response within the scaffolds. In Figure 12A, one can observe that samples 0, 2, and 5 maintained ALP activity levels below 10 U/L over the 14 days, indicating a relatively low level of osteogenic activity. However, these levels were notably higher compared to the activity levels found in samples S0 and S2, suggesting that incorporation of certain materials or modifications in samples 0, 2, and 5 may have had a positive impact on ALP activity, even without ultrasound irradiation. Conversely, when ultrasound irradiation was applied to nanofiber scaffolds seeded with MG-63 cells in samples S5 and S7, a significant increase in ALP activity was observed, almost doubling to approximately 20 U/L, as depicted in Figure 12B. This substantial enhancement in ALP activity highlights the potential of ultrasound irradiation in stimulating osteogenic differentiation and accelerating bone tissue regeneration. Notably, sample S7 exhibited particularly heightened ALP activity on day 14, indicating a sustained and intensified cellular response under the influence of ultrasound irradiation. Further analysis revealed that the presence of the TiO_2_ layer in the scaffold structure amplifies the chemical effects of ultrasound, enhancing cellular responses such as ALP activity. This synergistic effect between ultrasound irradiation and scaffold composition underscores the importance of tailored scaffold design and external stimuli in promoting osteogenic differentiation and tissue regeneration. The significant increase in ALP activity, a crucial osteogenic marker, holds promise for developing ultrasound-enhanced therapies aimed at accelerating bone tissue regeneration.^65^ These findings suggest that the combination of nanofiber scaffolds with ultrasound irradiation could serve as a promising strategy for improving the efficacy of bone tissue engineering applications.

## Conclusions

We designed a biocompatible and biodegradable scaffold based on PCL/PEG polymers with promising structural, mechanical, and biological properties. Its properties were further improved by incorporating mesoporous silica/titania nanoparticles loaded with CUR and BMP-2, and this CUR-BMP-2@DSTNs delivery system demonstrated controlled and sustained drug release capabilities which were further enhanced using low-intensity pulsed ultrasound.

All characterization techniques confirmed the successful integration of the CUR-BMP-2@DSTNs into the electrospun PCL/PEG nanofibers. The average diameter of the nanofibers was reduced by ca. 25%; their tensile strength increased by approximately 40% and their biodegradation degree by almost 20%, leading to a ca. 53% enhancement in their water absorption capacity. The SEM images of the deposited calcium/phosphate ceramics on the surface of the nanofibers showed that the osteoactivity of the nanofibers improved significantly, which were enhanced using ultrasound irradiation. Moreover, the nanofibers exhibited sustained and controlled release of the loaded drugs, which is crucial for effective bone tissue regeneration. To further enhance and control the release of the drugs, ultrasound was used, and we found that it had a positive impact on both aspects of drug release, leading to a controlled release of ca. 70% of the drug over 28 days. Furthermore, in vitro studies showed that the electrospun porous scaffolds closely mimic the natural extracellular matrix, providing an environment conducive to bone formation.

The biocompatible scaffold we designed and characterized in this paper is a compelling candidate for advanced bone tissue engineering and regenerative therapies. However, future works are required to better understand the effect of ultrasound as a promising technology on the properties of these biocompatible scaffolds.
